# Supplementary material for: Chromothripsis during telomere crisis is independent of NHEJ, and consistent with a replicative origin
Source: Genome Res. 2019 May;29(5):737–49. doi: 10.1101/gr.240705.118 (PMC6499312; doi:10.1101/gr.240705.118)
Supplement: Supplemental Material [file supp_gr.240705.118_Supplemental_file_1.zip › contigs/annotated_contigs/DB53/contig.8.DB53_length_1568_mean_cov_15.8718112245.docx]

**DB53_length_1568_mean_cov_15.8718112245**

```
GAGCCGGCAGGTCGCCCCATTACTTTTGTCTCTTTTGTTGTCATCTTAT|TTTACAACATGTTTCTCATTTAGATGTAATTTTCATTAT
                                                  >chr8:79037923-79038359 + E=6e-250
GTGATTAATATTATGCTATTTACTGTACATGTTTATATGCTTTTAGCTCTGCATGTGCAATATAAAATTAAAAGAAGTCTTTCCTCTTG

GAAATGTCAGTTTATTTTATTTTTTTTACTCTAAATCCAGAGTATGTTTTCAAGGTCTCTTCTAAAACGATAACCTTCATATGAGTCAT

AGGCATTTAAGTACATCTGTGGATTAGTCTGGCTTCAGTGAGGTTAAACGCAGGAAGAGTGTGCCTACAAAGAGATACGATAAGCAGTT

ATAATGGGTATCACTCAACACAGCACCAAATATAAATCAAAACTAACAAACACCCTCCCTCACAAATACTCTCAAGTGAGGTTCCTCAA

ATTTAATTATTACAGCAAATGGTAATGTAACAAGGGTGT|CT|CCCACAACCCCTTCCTCAAAAGTGTGAGTTGGGGGTGATTAGATGA
                                        >chr8:81612879-81612969 + E=5e-42
AGTAAGATGGACAAAATGTTAACAATTGTTGAAGCCAGGGAA|CCTCACTTGAGACAG|GTGAGCACATTTAAAGCACCACTCAG|AGG
                                                          >chr8:85468944-85468973 - E=
|CCCAGGAGTGACTGCAAGTGACACCTTCTCATATCCCAAATCACTCTGTCTGCAATAATTATACTAATATTATCTACTTCATGGGAGC
3e-05 >chr8:75096718-75097338 + E=0e+00
ATTGAGAACTGAGAGGTGGTGGGACAGAGGAAGTGTTCTAATTATTAGTAAGTCCAATTCCTACATATTTAAAAGGGACCATAAATGTT

TAACTATATGAAGGGAATTTGCAGATGTTTGTCTTTCAGTTGTGGAATCCCAGTTATGGGTGGTCTTTCACTCTGCTGTACATATTTGG

TCCATTTCCCTCAGTGCAATTTGGAACAGAGAGCATTCACTGAGTGCTGCTGTCTTTCTCCTGGAGATGGCTGTCCTGGCATCTGTCCT

TCCAGTTCTCCTCGACCCCCTGCACCCCACCCCTAATACACACACACACGAACTTCACTTTCTTTGCCTCCCTCCCTGGCTCTTTTTTC

TCCTTCCTCCTTTAGGCGAGGATCAAGGAGAAAGGAATTCCCTAGTTTCTTGCCCTGAGCTTCTCCAAACCCCGTCTGTGCTCTTGATC

TGTCCCATCCTGGGATCTCCACCTACTATTACCCTTACCATGAGCCCTTCAAACAGCCAACCTGTCAACTCAGCTTCGCC|TTG|TTTT
                                                                                 >chr8:
TGAAGAATGCAAAGTAAAAAGAAAGGAGAAGAACCTAGCATGACCAGAACACCTACTCTATACCAAACAGGGCACTCAGGGTTTTTAAT
85360907-85361142 - E=4e-129
CCATAGAGGGAATGGACATACTCAAAGCTCATATATCCCAAGATTTTTACGCTATAAGAGGTGAGGTCCTAAAAACAAAATGTATGAAT

CTCTATGAGTCAAAAAAGACAGCTACTTGTTCTCCTTGTCTTCATTTTGC|TGGCACAGCCATAGTTGCTGAAGAATCCTGCTTACATT
                                                    >chr8:79152402-79152447 - E=6e-15
AAAAC|AT|GTCAGTGACACCAAGGTTCTAAAA|CT|GTACAGCACGTTACTGTACTGAATACTGTAG|G      >chr17:68800146-68800174 - E=1e-04  >chr4:109185936-1091859
```
